# The changing epidemiology of varicella and herpes zoster in Hong Kong before universal varicella vaccination in 2014

**DOI:** 10.1017/S0950268818000444

**Published:** 2018-03-12

**Authors:** D. Y. W. Chan, W. J. Edmunds, H. L. Chan, V. Chan, Y. C. K. Lam, S. L. Thomas, A. J. van Hoek, S. Flasche

**Affiliations:** 1 Communicable Disease Division, Surveillance and Epidemiology Branch, Department of Health, Centre for Health Protection, Hong Kong, Hong Kong SAR; 2 Department of Infectious Disease Epidemiology, Faculty of Epidemiology and Population Health, London School of Hygiene and Tropical Medicine, London, UK

**Keywords:** Chickenpox, surveillance, vaccines, varicella zoster, zoster (shingles)

## Abstract

In Hong Kong, universal varicella vaccination started in July 2014. Before this, children could receive varicella vaccine via the private market. We analysed the epidemiology of varicella and zoster before universal vaccination. We estimated varicella vaccination coverage through surveys in preschool children. We estimated the burden of varicella and zoster with varicella notifications from 1999/00 to 2013/14, Accident and Emergency Department (A&E) attendance and inpatient admissions to public hospitals from 2004/05 to 2013/14. We fitted a catalytic model to serological data on antibodies against varicella-zoster virus to estimate the force of infection. We found that varicella vaccination coverage gradually increased to about 50% before programme inception. In children younger than 5 years, the annual rate of varicella notifications, varicella admission and zoster A&E attendance generally declined. The annual notification, A&E attendance and hospitalisation rate of varicella and zoster generally increased for individuals between 10 and 59 years old. Varicella serology indicated an age shift during the study period towards a higher proportion of infections in slightly older individuals, but the change was most notable before vaccine licensure. In conclusion, we observed a shift in the burden of varicella to slightly older age groups with a corresponding increase in incidence but it cannot necessarily be attributed to private market vaccine coverage alone. Increasing varicella vaccination uptake in the private market might affect varicella transmission and epidemiology, but not to the level of interrupting transmission.

## Introduction

Varicella is an endemic disease in most parts of the world and it is the most commonly reported notifiable infectious disease in Hong Kong [1]. Varicella vaccination has been effective in reducing the disease burden of varicella wherein routine use [2–5]. However, vaccination at low coverage may not lead to an interruption of transmission, and it may even contribute to a shift of the varicella burden in unprotected individuals towards older ages who are typically at higher risk for severe outcomes [6]. Vaccines against varicella and herpes zoster have been available in Hong Kong through the private market since 1996 and 2006, respectively. Varicella vaccine was included in the Hong Kong Childhood Immunisation Programme (HKCIP) in July 2014 for children born on or after 1 January 2013 [7]. Under the HKCIP, eligible children receive monovalent varicella vaccine (mVV) at 12 months of age and combined MMRV (measles, mumps, rubella and varicella vaccine) during their first year at primary school (about 6 years of age) as a second dose.

We document the varicella vaccination uptake before universal childhood vaccination alongside serological population profiles and the disease burden of varicella and zoster. We describe the burden of varicella and zoster in Hong Kong in the pre-universal varicella vaccination era and its changing epidemiology as a result of varicella vaccination through the private market.

## Methods

### Varicella vaccination coverage in pre-school children

The Department of Health (DH) conducted six rounds of territory-wide immunisation surveys to assess the uptake of different vaccines in preschool children in 2001 [8], 2003 [9], 2006 [10], 2009 [11], 2012 [12] and 2015 [13]. In summary, pre-schools in Hong Kong were selected using stratified cluster sampling. About 5% of preschools were selected in each survey (range: 24–71). All parents of the selected preschools were invited to join the survey. Consented parents completed a self-administered questionnaire to collect demographic information on children (including place of birth and residence) and provide all relevant immunisation records to us through the pre-primary institutions. During field visits, trained field workers collected the questionnaires and extracted information from the immunisation records to a standardised data-recording form. When the immunisation record was incomplete, parents were asked to provide other immunisation documents, if any. The response rate of these six surveys was at least 73% [8–13]. There is a high rate of pre-school attendance amongst children aged 3–5 years in Hong Kong [14]. Thus, we included children aged 3–5 years in each survey for analysis and excluded younger children. Before 2015 only information for the first dose was recorded for vaccines not included in the routine immunisation schedule, including varicella vaccines. Date of vaccination was only collected in the 2015 survey. We defined varicella vaccination uptake as the proportion of respondents who received at least one dose of varicella vaccine.

### Disease burden of varicella and zoster

We used data of varicella notifications to the DH from 1999 until 2014 which included reports from public and private health sector physicians and schools. The clinical case definition for such notifications is the acute onset of diffuse (generalised) papulovesicular rash without other apparent cause or alternatively atypical (milder) clinical presentation with previous varicella vaccination. We defined probable cases as those fulfilling the clinical case definition and confirmed cases as probable cases with either laboratory confirmation or with known epidemiological links to a confirmed case [15]. We included all confirmed and probable cases in our analyses.

Furthermore, we used data on Accident and Emergency Department (A&E) attendance and inpatient admissions to public hospitals routinely collected via the Communicable Disease Information System (CDIS) of the DH. In Hong Kong, public hospitals account for around 80% of all hospitalisations [16]. Doctors assign ICD-9-CM diagnosis codes for A&E attendance and inpatient admissions as part of the clinical record. The main condition contributing to the disease episode is assigned as the primary (principal) diagnosis code, whereas other contributing condition(s) are assigned as secondary diagnosis codes. We included all A&E and inpatient admission records with an ICD-9-CM code of varicella (052) and/ or zoster (053) in the primary or secondary diagnosis code. Data from 2004 to 2015 were available for analyses. In addition, we analysed principal and secondary diagnosis codes to record the frequency of other common complications related to varicella and zoster, presence of immune-compromising conditions and pregnancy, according to a pre-defined list of ICD-9-CM codes (Table S1). All varicella and zoster attendance and admissions after the initial diagnosis were assumed to be part of the same illness episode. We defined the length of stay for inpatients with multiple varicella or zoster admissions during one illness episode as the cumulative number of days in the hospital. Fatal cases were defined based on discharge information in the last admission episodes.

In the fiscal year of 2009/10, the Hospital Authority (HA) introduced a pay-for-performance funding model to tie budget allocations with services provided by different hospitals [17]. In this system, the diagnoses codes are used to measure workloads [17]. We observed changes in coding practice during our study period as coding was more complete after 2009/10 (Fig. S1). Therefore, changes in the rate of varicella and zoster A&E attendance and admission might be affected by the improvement in coding completeness. To account for this change, we adjusted the annual number of A&E attendances (hospital admissions) that were coded as varicella according to the principal diagnosis by the proportion of attendances (admissions) not coded in the corresponding year and age group relative to the last study year (2013/14) (Fig. S1). Similarly, we adjusted the annual number of attendances (admissions) that were coded as varicella according to the secondary diagnoses by the average number of codes assigned per attendance (admission) in the corresponding year and age group relative to the last study year. We similarly adjusted for a change in coding for zoster.

We defined an epidemiological year as the 12 months from September to August to account for the seasonality of varicella disease burden in Hong Kong. We obtained annual age-stratified population estimates from the Census and Statistics Department [18] to compute the annual rate of varicella notification, A&E attendance and hospitalisation. We conducted Poisson (log-linear) regression to evaluate trends in age-specific annual incidence rates for different surveillance data:

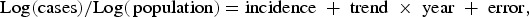
where the population was included as an offset, year indicated the number of years before 2013/14 and coefficient (trend) indicated the annual change in rate with a value of 1 equivalent to no trend.

### Varicella serology and force of infection

The Public Health Laboratory Services Branch (PHLSB) of the DH conducted serological surveillance on antibodies against varicella-zoster virus (VZV) in 1995, 2000, 2005 and 2010 [19]. Convenience samples were selected from sera submitted for laboratory tests other than virology. These residual sera were collected until a predefined number was attained for different age groups. To assess whether increasing vaccine uptake in the private market had impacted varicella transmission over the study period, we fitted a single catalytic model [20] to the seroprevalence data of all 4 years in order to estimate potential changes in the force of infection (FOI – the annual rate at which susceptible individuals become infected). We assumed that infants younger than six months are protected by maternal antibodies. We found that similar to other studies, more than 90% of adults in our study had seroconverted at the time of testing [21]. Hence, we assumed individuals aged 20 years or older contribute little to transmission and restricted the fitting of our model to data from children. Since only data for four broad age strata were available for individuals under 20 years old [19], we estimated and compared the overall FOI for these individuals in different periods. We further assumed that the FOI was constant before 1995, and we estimated potential changes in the force of infection between later surveys. We jointly estimated those FOIs using a Metropolis–Hastings Markov Chain Monte Carlo algorithm with a binomial likelihood. The model equation reads:

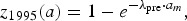


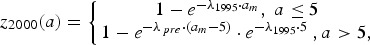


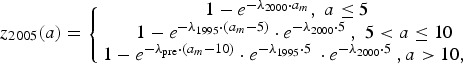


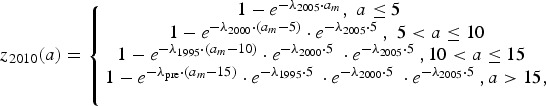
where *z*_*Y*_(*a*) is the proportion of seropositive individuals in year *Y* and at age *a* and *a*_*m*_ = *a* − 0.5. Further, *λ*_*pre*_, *λ*_1995_, *λ*_2000_, *λ*_2005_ are the FOI before 1995, from 1995 to 1999, from 2000 to 2004 and from 2005 onwards, respectively.

As varicella vaccine was available in the private market since 1996 and the vaccine uptake increased over the years, some individuals tested seropositive might have seroconverted through vaccination irrespective of natural infection. To adjust for that we calculated the age-specific proportion of vaccinees in respective survey years by interpolating results from the coverage surveys, assuming 65% of them seroconverted [22] (Table S2) and the remaining proportion of the population could seroconvert due to natural infection:

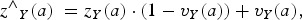
where *z*^_*Y*_(*a*)is the adjusted proportion of seropositive individuals in year *Y* and at age *a*, after taking into account the proportion of seroconversion arising from vaccination (*v*_*Y*_(*a*)) and *z*_*Y*_(*a*) as defined above.

We conducted a sensitivity analysis on the model fitting by using data estimated from the 95% confidence bounds of the proportion of vaccinees seroconverted (Fig. S3). Based on the FOI estimated, we computed the reproduction number corresponding to each estimated FOI by assuming random mixing and a rectangular age distribution as *R*_0_ = *L* · *λ* [23], where *L* is the average life-expectancy in Hong Kong in 1995 [24].

We further estimated the number of new infections *I*_*Y*_(*a*) as the product of the proportion of seronegative (and hence susceptible), the annual FOI and the respective population size (*P*_*Y*_(*a*)):

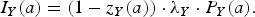


To assess underreporting of varicella notification, we computed the ratio between the number of notifications and the estimated number of new infections. As age-specific notification data are not available for 1995, this analysis was limited to 2000, 2005 and 2010 only.

All statistical tests and the catalytic model were programmed in R [25].

## Results

### Varicella vaccination uptake in preschool children

**Fig. 1.**
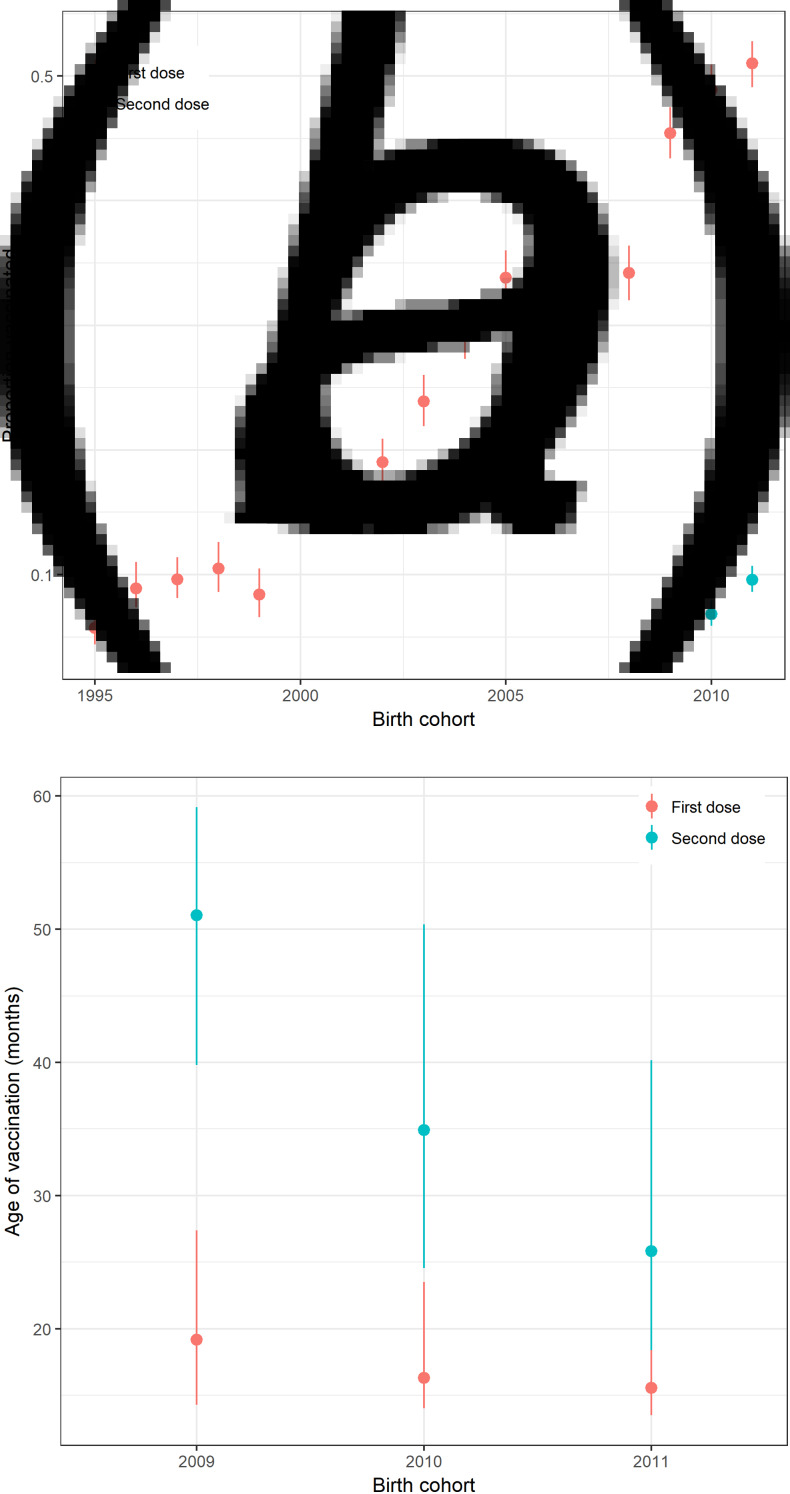
Varicella vaccination in preschool children in Hong Kong. (a) Proportion of pre-school children in Hong Kong receiving varicella vaccine by birth cohort, 1995–2011 and (b) Interquartile range of age at receipt of varicella vaccination (months) for preschool children born from 2009 to 2011 by birth cohort and a dose of vaccine calculated from the survey in 2015. Note: Uptake on the second dose of vaccine was only recorded for children born in 2009–2011.

Uptake of at least one dose of varicella vaccine among preschool children gradually increased to about 50% during the decade before the introduction of the universal varicella vaccination programme in Hong Kong (Fig. 1a). For children born in 2009, 2010 and 2011, <10% had received a second dose of varicella vaccine before the age of 6, 5 and 4 years, respectively. Most children in these cohorts received the first dose of varicella vaccine before 20 months of age (Fig. 1b).

### Disease burden, temporal trends and seasonality of varicella and zoster

**Fig. 2.**
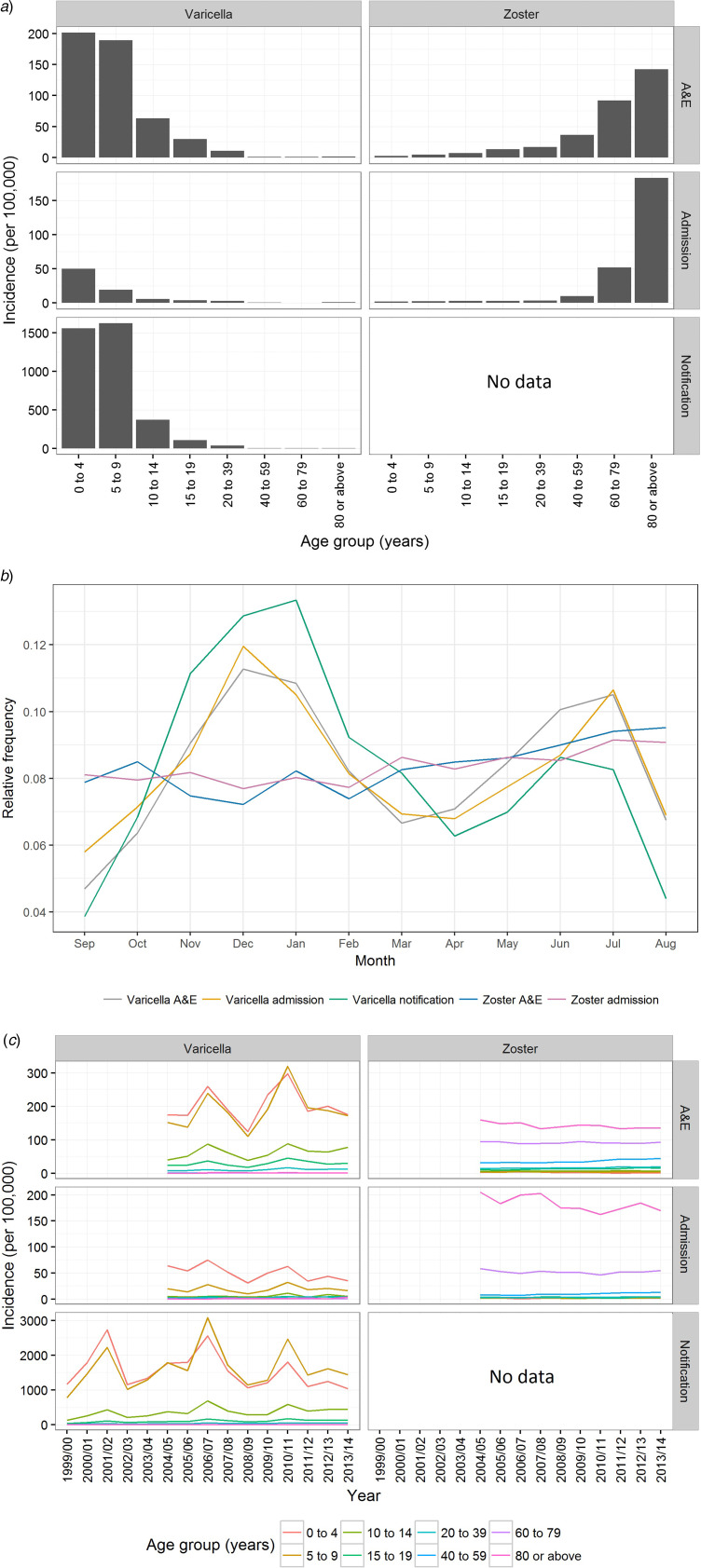
Varicella notification, varicella and zoster A&E attendance and hospitalisation in Hong Kong. (a) The average annual rate of varicella and zoster in Hong Kong during the study period, (b) relative frequency distribution for cases of all ages by month throughout the study period and (c) rate of varicella and zoster by epidemiological year. Notification is available only for varicella. Epidemiological year was defined as 12 months from September to August.

From September 1999 to August 2014, over 173 000 varicella cases were reported to the DH, corresponding to an average annual incidence of 156 per 100 000 population. There were 14 144 varicella episodes recorded under A&E attendance and 2860 under inpatient admissions during the same period (Table 1). Varicella primarily affected young children aged under 10 years. The average annual notification, A&E attendance and inpatient admission rate decreased with age (Fig. 2a). The average annual notification rate per 100 000 for those aged 5–9 years and those aged under 5 years were similar (1620 and 1555, respectively). On the other hand, the A&E attendance and inpatient admission were highest for those aged under 5 years (202 and 51 per 100 000, respectively).

**Table 1.** Baseline characteristics of varicella and zoster cases in Hong Kong

		Varicella			Zoster	
		Statutory notification	A&E attendance	Inpatient admission	A&E attendance	Inpatient admission
Study period		September 1999–August 2014	September 2004–August 2014			
No. of cases		173 748	14 144	2860	23 456	12 885
No. of cases with multiple attendance/admission (%)		NA	1470 (10)	164 (6)	4423 (19)	2600 (20)
No. of attendance/admission	Median (range)	NA	1 (1–6)	1 (1–11)	1 (1–48)	1 (1–37)
Gender (%)	Male	90 355 (52)	7626 (54)	1546 (54)	11 721 (50)	6066 (47)
	Female	80 391 (47)	6518 (46)	1314 (46)	11 735 (50)	6819 (53)
	Unknown	2002 (1)	0 (0)	0 (0)	0 (0)	0 (0)
Age in years	Median (range)	6 (0–99)	7 (0–92)	6 (0–98)	59 (0–104)	73 (0–110)
CFR (per 1000 cases)		0	0	3	0	38

Four per cent of cases admitted due to varicella were immunodeficient (Table 2). Nineteen per cent of female admissions aged 20 years or above were pregnant. Less than 1% of varicella A&E attendance was associated with complications, compared with 20% in varicella admission (Tables 2).

**Table 2.** Underlying medical conditions, complications and outcomes of varicella-related A&E attendance and inpatient admissions

	A&E attendance in public hospitals		Inpatient admission in public hospitals	
	*N*	%	*N*	%
No. of cases	14 144	–	2860	–
Underlying medical conditions				
Pregnancy^a^	15	1.2	87	19.0
Immunodeficiency (any)^b^	0	–	110	3.8
Lymphoproliferative malignancy	0	–	50	1.7
Malignancy of solid organ/tissue	0	–	21	0.7
Transplant	0	–	22	0.8
HIV	0	–	5	0.2
Other immunodeficiency condition^c^	0	–	25	0.9
Complication^b^	97	0.7	575	20.1
Pneumonia	12	0.1	92	3.2
*Varicella pneumonitis*	4	0.0	10	0.3
*Other pneumonia*	8	0.1	82	2.9
Neurological disorder	6	0.0	35	1.2
*Encephalitis*	0	–	12	0.4
*Ataxia*	0	–	10	0.3
*Meningitis*	6	0.0	13	0.5
*Myelitis*	0	–	2	0.1
Other unspecified viral infection of the central nervous system	0	–	0	–
Septicaemia/sepsis	0	–	21	0.7
Bacterial infections (including scarlet fever and bacterial infection caused by Streptococcal and Staphylococcal species)	0	–	238	8.3
Bacterial superinfection of skin and/or soft tissue	14	0.1	101	3.5
*Cellulitis*, *abscess* and *erysipelas*	13	0.1	83	2.9
*Impetigo*	1	0.0	17	0.6
*Fasciitis*	0	–	3	0.1
Ophthalmic disorders (including conjunctivitis and keratoconjunctivitis)	11	0.1	14	0.5
Congenital varicella infection	0	–	0	–
Outcome				
Hospitalisation	563	4.0	NA	NA
Median length of stay (days)	NA	NA	4	
Among cases with complications	NA	NA	5	
Among cases without complications	NA	NA	4	
ICU admission	NA	NA	19	0.7
Among cases with complications	NA	NA	6	1.0
Among cases without complications	NA	NA	13	0.6
Death	0	0.0	9	0.3
Among cases with complications	–	–	6	1.0
Among cases without complications	–	–	3	0.1

a Pregnancy among females aged 20 years or above.

b More than one type of immunodeficiency/complications were coded in some episode of A&E attendance and admission.

c These include conditions such as white blood cell diseases, Thalassaemia major, aplastic anaemia, asplenia or other splenic diseases, other specified diseases with the participation of lymphoreticular and reticulohistiocytic tissue, patients undergoing chemotherapy or radiotherapy and other conditions affecting the immune system.

The number of zoster A&E attendance and inpatient admissions was higher than that for varicella. Nineteen per cent had multiple A&E attendance or admissions (Table 1). The median age of zoster A&E attendance and admissions were 59 and 73 years, respectively. In contrast to varicella, the average annual A&E attendance and inpatient admission rate for zoster increased with age (Fig. 2a) and was highest for those aged 60 years and above (both 117 per 100 000). The rate of zoster admission for those aged 80 years and above was nearly four times higher than that for those aged 60–79 (Fig. 2a). Complications occurred in 23% of zoster admissions, with post-herpetic neuralgia (PHN) (13%) and ophthalmic complications (5%) being the most common (Table 3).

**Table 3.** Underlying medical conditions, complications and outcomes of zoster-related A&E attendance and inpatient admissions

	A&E attendance in public hospitals		Inpatient admission in public hospitals	
	*N*	%	*N*	%
No. of cases	23 456	–	12 885	–
Underlying medical conditions				
Pregnancy^a^	3	0.0	38	0.6
Immunodeficiency (any)	11	0.0	1952	15.1
Lymphoproliferative malignancy	2	0.0	691	5.4
Malignancy of solid organ/tissue	5	0.0	855	6.6
Transplant	0	0.0	259	2.0
HIV	3	0.0	68	0.5
Other immunodeficiency condition^b^	1	0.0	303	2.4
Complication	426	1.8	2989	23.2
Neurological disorders	1920	8.2	2047	15.9
*Encephalitis*	2	0.0	28	0.2
*Meningitis*	14	0.1	61	0.5
*Post herpetic neuralgia*	1848	7.9	1646	12.8
*Post herpetic trigeminal neuralgia*	35	0.2	65	0.5
*Post herpetic polyneuropathy*	8	0.0	8	0.1
*Viral infection of the nervous system*	22	0.1	72	0.6
Ophthalmic complications	699	2.9	627	4.9
Geniculate zoster/Ramsay-hunt syndrome	318	1.4	400	3.1
Auricular involvement	31	0.1	37	0.3
Disseminated zoster	13	0.1	77	0.6
Outcome				
Hospitalisation	1485	6.3	NA	NA
Median length of stay (days)	NA	NA	7	
Among cases with complications	NA	NA	8	
Among cases without complications	NA	NA	6	
ICU admission	NA	NA	24	0.2
Among cases with complications	NA	NA	7	0.2
Among cases without complications	NA	NA	17	0.2
Death	0	0.0	484	3.8
Among cases with complications	–	–	117	3.9
Among cases without complications	–	–	367	3.7

a Pregnancy among females aged 20 years or above.

b These include conditions such as white blood cell diseases, Thalassaemia major, aplastic anaemia, asplenia or other splenic diseases, other specified diseases with the participation of lymphoreticular and reticulohistiocytic tissue, patients undergoing chemotherapy or radiotherapy and other conditions affecting the immune system.

There were no deaths recorded for varicella and zoster A&E attendance. The crude case-fatality rate of varicella notification, varicella and zoster admissions were 0.005, 3.1 and 37.6 deaths per 1000 cases, respectively.

Varicella notifications, A&E attendance and hospitalisations exhibited strong seasonal patterns with additional inter-season variation (Figs 2b & c). In contrast, zoster-related A&E attendance and admission did not show much intra- or inter-season variance (Figs 2b & c). Both the rate of zoster A&E attendance and admission increased with age.

### Changing epidemiology over time

**Fig. 3.**
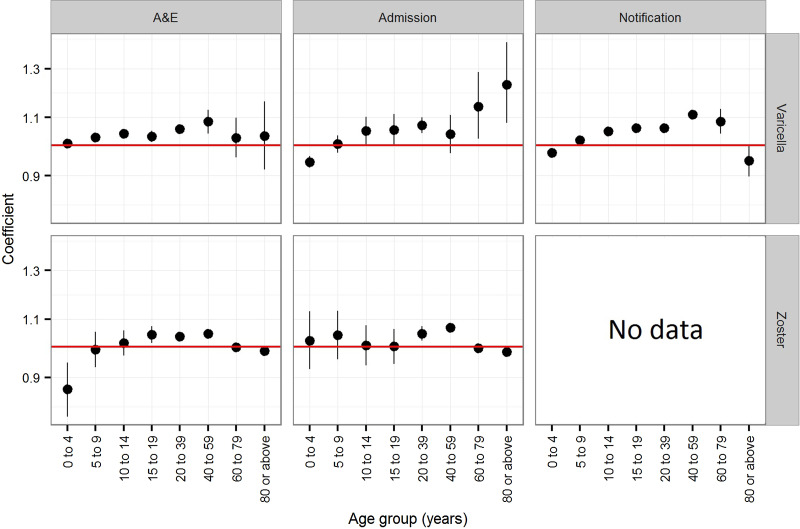
Coefficients (trends) of the Poisson regression on the annual rate of varicella notification, varicella and zoster A&E attendance and hospitalsations in Hong Kong. Notification is available only for varicella. A coefficient of zero indicates no change.

The estimated coefficients for time trend in the annual rate of varicella notification, varicella and zoster A&E attendance and hospitalisations in Hong Kong is presented in Figure 3. In children younger than 5 years, the annual rate of varicella notification (−2.5% (95% CI −2.4 to −2.7%)), varicella admissions (−5.6% (95% CI −3.7 to −7.5%)) and zoster A&E attendance (−13.3% (95% CI −5.3 to −21.3%)) generally decreased during the study period (Fig. 3). The annual notification, A&E attendance and hospitalisation rate of varicella and zoster generally increased for individuals between 10 and 59 years old, although no significant changes were observed for some age groups (those aged 10–14 years in zoster A&E attendance, those aged 40–59 years in varicella admission and those aged 10–19 years in zoster admission). The annual rate of increase was 3.0% (95% CI 1.1–5.0%) to 11.2% (95% CI 9.5–12.9%) for varicella and 0.2% (95% CI −5.7 to 6.3%) to 6.8% (95% CI 5.3–8.3%) for zoster. For children aged 5–9 years and adults of at least 60 years, we found no consistent trend in either direction.

**Fig. 4.**
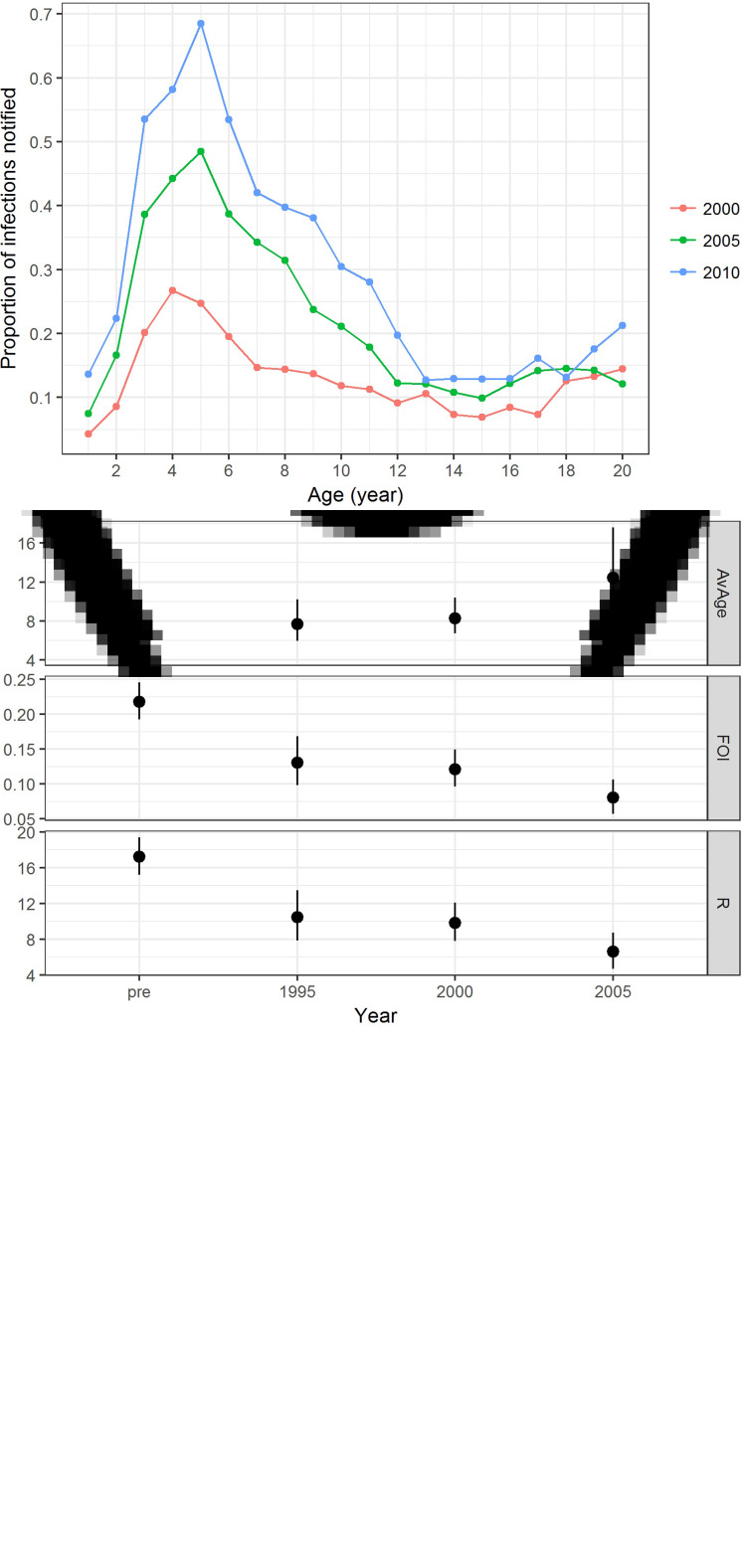
Varicella serology and estimated transmission parameters in Hong Kong in 1995, 2000, 2005 and 2010. (a) Proportion seropositive against varicella antibody by ELISA test (points with error bars representing 95% CI) and model fitting (line charts with shaded errors bands representing 95% CI), (b) average age of infection [AvAge], annual average force of infection [FOI] and basic reproduction number [R] and (c) reporting ratio between varicella notification and number of infections estimated. Age-specific data on varicella notification are only available for analysis for the year 1999 and onwards.

In addition to varicella notification, varicella serology also indicated an age shift towards a higher proportion of infections in slightly older individuals. These changes were most notable from the period before vaccine licensure to vaccine introduction in the private market and when the vaccine uptake further increased in the private market from 2005 onwards (Fig. 4a & b). The annual FOI before vaccine introduction in the private market was estimated to be 0.22 (95% CI 0.19–0.25) and it decreased to 0.13 (95% CI 0.10–0.17) from 1995 to 1999. After vaccine licensure, the FOI from 2000 to 2004 was stable at 0.12 (95% CI 0.10–0.15), but it further reduced to 0.08 (95% CI 0.06–0.11) from 2005 onwards (Fig. 4b). This corresponds to a reduction in reproduction number from 17.2 (95% CI 15.2–19.4) to 10.4 (95% CI 7.8–13.5), 9.8 (95% CI 7.8–12.1) and 6.6 (95% CI 4.7–8.7) (Fig. 4b). The estimated average age of infection increased from 4.6 (95% CI 4.1–5.2) in 1995 to 7.7 (95% CI 5.9–10.2) in 2000, and 8.3 (95% CI 6.7–10.4) in 2005 and 12.4 (95% CI 9.4–17.6) in 2010.

### Underreporting of varicella notification

When compared with the number of varicella notifications, 13%, 22% and 29% of the estimated varicella infections in 2000, 2005 and 2010 were reported. The reporting ratio differed by age and year (Fig. 4c). Reporting was most complete for those aged 3–10 years.

## Discussion

Before the introduction of universal varicella vaccination in Hong Kong, vaccine uptake through the private market increased gradually to about 50%. We showed that varicella and zoster are common diseases in Hong Kong. We observed a shift in the burden of varicella to slightly older age groups with a corresponding increase in A&E attendance and hospitalisations in these age groups. We also showed from cross-sectional serological surveys that the transmission intensity of varicella decreased while the average age of varicella infection increased shortly after vaccine licensure. Therefore, the shift in disease burden may not be solely associated with the increasing vaccine uptake in the community.

The annual FOI before vaccine use in the private sector was estimated to be 0.22 (95% CI 0.19–0.25), which is high compared with the FOI in Australia before use of varicella vaccine [26] and comparable with some of the estimates from 11 countries in Europe [21]. The difference in the intensity and pattern of population mixing may contribute to the variations in varicella transmission estimates. It should be noted that varicella transmission may be facilitated in a densely populated area. The population density (per square kilometre) in Hong Kong (5758) was much higher than those in Australia (2) and the 11 countries included in the European study (15–378), according to estimates of United Nations in 1995 [27].

The decrease in transmission intensity of varicella was most prominent from 1995 to 1999, but the initial vaccine uptake during this period was low. While the low vaccination uptake might have had some effect on varicella transmission, other factors such as temporal changes in contact mixing could have played an additional role. However, the first published social contact data in Hong Kong were collected in 2009 and 2010 [28] and earlier data are not available for comparison. Nevertheless, we did observe some reduction in the overall number of students and class size in a comparable period from data from the Education Bureau [29] and Social Welfare Department [30] (Fig. S2).

The age shift in varicella infection leaves unvaccinated individuals at increased risk of complications that accompany infection [31]. This includes varicella infection during pregnancy which can result in transplacental transmission of VZV to the foetus and lead to stillbirths or permanent congenital defects [32]. Our analysis of public hospital admissions showed that 19% of women aged 20 years or older with varicella-related admissions were pregnant. This overrepresentation of pregnant women further highlights the need for increased awareness among healthcare workers of the risk of varicella during pregnancy.

Albeit inconclusive from this analysis, if indeed the increased vaccine uptake in the private sector contributed to the age shift in varicella burden, it creates an issue of equity as those older non-immune children not eligible for universal vaccination would be less likely to acquire natural immunity at a young age. With the inclusion of varicella vaccine in the Childhood Immunisation Programme, vaccination uptake should soon exceed 90% and, in the mid-term, likely prevents the majority of varicella illnesses during childhood. However, in the short-term the additional reduction in transmission leaves those cohorts of children who are too old to be eligible for vaccination and who have not been vaccinated through the private market at an even higher risk. We have established a baseline rate which will allow monitoring to identify trends in varicella complications and act on them swiftly.

Furthermore, we found that the burden of zoster A&E attendance and hospitalisations in adults has been on the rise in the pre-universal varicella vaccination era. Circulation of VZV in the community is believed to boost immunity against zoster for individuals previously infected with varicella. It is possible that the gradual increase in varicella vaccination uptake reduced transmission and hence boosting, leading to the observed increase in zoster A&E attendance and hospitalisations among adults. However, the estimated annual decrease in varicella notification and admission for children was 2.5% and 5.6%, and hence effects on transmission may be relatively small. The relationship of childhood varicella vaccination and adult zoster burden continues to be a point for discussion [33]. In addition, other temporal changes might have also contributed to this increase in burden (Fig. S2). In particular, the change in the incentive to code A&E attendance and hospital admissions has made such interpretation difficult. While we accounted for such changes in coding for both primary and secondary diagnoses when estimating the burden of A&E attendance and hospitalisations, we cannot rule out that such changes have contributed to our findings of increasing rate of zoster A&E attendance and hospitalisations among adults.

Based on the existing data, we found no evidence of an increase in zoster A&E attendance and hospitalisation rate among the two oldest age groups, for which the burden of zoster disease is highest. However, this observation may have been affected by a change in healthcare-seeking behaviour instead of zoster vaccination, for which <10% of those aged 60 years or above should have received zoster vaccination (personal communication with Drug Office of the Department of Health). Specifically, since 2009 the Government introduced Health Care Vouchers (HCV) for elders aged 70 years or above to subsidise healthcare costs in the private medical sector (which is not covered by our zoster data) [34]. The amount of annual subsidy has increased from $250 in 2009 to $2000 in 2015. Cross-sectional surveys showed that the proportion of the elderly having ever used HCV increased from 35% in 2010 [35] to 80% in 2015 [36]. Thematic Household Surveys [16] conducted by the Census & Statistics Department indicated that individuals aged 65 years or above attended more private practitioners but less A&E doctors in recent years (personal communication with Census & Statistics Department). The proportion of inpatient admission at private hospitals increased from 4% in 1999 to 10% in 2014.

Based on the number of varicella infections estimated by fitting the serological data to the catalytic model, only 13–29% of infections were notified. This is comparable with studies in Italy [37] and the USA [38], which similarly found that notification was most complete in children 5–9 years old and that less than half of varicella cases are notified, albeit using different methodologies to estimate underreporting. Varicella is a generally mild disease and hence parents may be reluctant to seek medical consultation. In particular, Hong Kong parents do not need medical confirmation if their child is absent from (pre-) school. Furthermore, doctors may mis-classify particularly mild symptoms of varicella. Reporting was most complete in children 3–6 years old in our study. Under the current reporting system, (pre-) schools are required to report varicella outbreaks and hence cases that are not usually seen by a doctor could be identified this way. We also found that reporting was more complete in later years, potentially related to increased awareness of varicella after the vaccine was more widely available in the community.

There are several limitations of this study. First, A&E attendance and admission with diagnosis codes for VZV in pregnancy/postpartum were not available and this may lead to slight underestimation of the disease burden. Second, clinical notes were not available to ascertain the specificity and accuracy of the ICD codings. For instance, there were one and 21 A&E attendance and hospitalisations coded with both varicella and zoster, respectively. Likewise, fatal outcomes following varicella and zoster A&E attendance and hospitalisations might be attributed to other concurrent diseases, which resulted in an apparently high case-fatality rate of zoster. On the other hand, only 4% and 17% of the hospitalised varicella and zoster cases had codes related to immune-deficient conditions (Table S1), which were lower than one US study using similar methodology [39]. This could be due to the difference in coding practice or criteria in admitting cases, but it is difficult to ascertain without review of clinical records. Third, although we adjusted the estimates of varicella and zoster A&E attendance and admissions rate with the coding rate in respective years, changes in healthcare-seeking behaviour might vary during the study period. Fourth, residual sera for the seroprevalence surveys were sampled conveniently and they might differ from the general population, although a study in Australia [40] showed that sero-immunity against most vaccine-preventable diseases estimated from samples collected through convenient and random sampling were largely comparable. Fifth, while commercially available VZV whole-cell (wc) enzyme-linked immunosorbent assay (ELISA) kits are regarded as sufficient in detecting seroconversion arising from natural infection, its reliability in assessing seroconversion from vaccination is not well documented [41]. In our model, we rely on an estimate from Sauerbrei *et al.* [22] for which 37 of 57 (65% [95% CI 51–77%]) of individuals aged 2–35 years were tested positive by a commercially available wc ELISA kit 4–6 weeks after receiving one or two doses of mVV. We conducted sensitivity analysis on model fitting based on data estimated from the confidence bounds of Sauerbrei *et al.*’s work. This showed that the modelled proportion seropositive was largely comparable even after taking into account variations in the seroconversion among vaccinees (Fig. S3). In addition, circulation of wild-type VZV is believed to boost immunity of previously infected individuals [42]. There is also evidence of such boosting in the persistence of immunity in vaccinees [43]. Therefore, natural infection (as indicated by the FOI) might not only contribute to the seroconversion in those naturally infected but also the vaccinees, especially a few years after vaccination. This is particularly relevant to our study in Hong Kong, as the circulation of VZV continues among those unvaccinated in the absence of universal vaccination. The magnitude of such boosting in vaccinees is not well understood. Sixth, consultations in public and private outpatient settings account for nearly 80% of all consultations [16], but zoster data in these settings were not available for a more comprehensive assessment of the disease burden. Furthermore, non-residents who sought medical care in Hong Kong may contribute to the burden of varicella and zoster as well as varicella serology. Importation status was only available for varicella notifications from 1999 to 2003, but imported cases contributed to only 0.2% of all notifications in this period. This proportion might have increased in recent years as the number of babies born in Hong Kong to mainland women was on the rise until 2013 [44]. Although some of these babies are raised in mainland China, they may later move to Hong Kong for education or continue to reside in mainland China but travel to Hong Kong regularly as cross-border students [45]. The impact of these demographic changes to our observations is unclear as details are not available.

In conclusion, we establish an estimate of the burden of varicella and zoster in Hong Kong in the era before universal childhood vaccination against varicella. We show that vaccination uptake increased substantially through private sector access before universal vaccination; although not to levels that likely interrupt transmission. We present evidence that during the same time period, the disease burden of varicella has shifted towards older children which sees unvaccinated individuals at increased risk of disease complications. Concurrently we observed an increase in zoster A&E attendance and hospitalisation rate among young and middle-aged adults. Serological data extending back to 1995 suggest that age shifts in varicella infections started before licensure of varicella vaccine and hence cannot necessarily be attributed to private market vaccine coverage alone. With the decision taken in 2013 to offer universal varicella vaccination in Hong Kong, we expect the burden of varicella in young children to decline in subsequent years. However, further research is needed to appraise the necessity of a catch-up campaign to protect children not currently eligible for vaccination, the likely impact of different vaccination schedules, and the cost-effectiveness of the programme.

## Supplementary material

For supplementary material accompanying this paper visit https://doi.org/10.1017/S0950268818000444. click here to view supplementary material
