# Supplementary material for: The changing epidemiology of varicella and herpes zoster in Hong Kong before universal varicella vaccination in 2014
Source: Epidemiol Infect. 2018 Mar 12;146(6):723–34. doi: 10.1017/S0950268818000444 (PMC6533643; doi:10.1017/S0950268818000444)
Supplement: Supplementary file 1 [file S0950268818000444sup001.docx]

**Epidemiology and Infection**

**Title: The changing epidemiology of varicella and herpes zoster in Hong Kong before universal varicella vaccination in 2014**

**Authors**

**D.Y.W. CHAN, W. J. EDMUNDS, H.L. CHAN, V. CHAN, Y.C.K. LAM, S.L. THOMAS, A. J. VAN HOEK, S. FLASCHE**

**Supplementary Material**

Supplementary Table S1. ICD-9-CM codes related to disease and common complications of varicella and zoster, as well as underlying conditions of pregnancy and immunodeficiency

Supplementary Table S2. Estimation of proportion seroconverted among vaccinees in different serological surveys

Supplementary Figure S1. Coding practice of A&E attendance and inpatient admission in public hospitals of Hong Kong, 2004/05 to 2013/14. (a) Proportion of A&E attendance coded, (b) average number of codes per A&E attendance, (c) proportion of A&E referred admission coded and (d) average number of codes per A&E referred admission

Supplementary Figure S2. No. of enrolment, average no. of enrolment per school and per class in preschools^, primary and secondary schools in Hong Kong, 1990 to 2015 (Source: Education Bureau and Social Welfare Department, Hong Kong SAR Government).

Supplementary Figure S3. Proportion seropositive against varicella antibody and model fitting with sensitivity analysis.

**Supplementary Table S1. ICD-9-CM codes related to disease and common complications of varicella and zoster, as well as underlying conditions of pregnancy and immunodeficiency**

| **Condition** | **Description** | **ICD-9-CM Codes** |
| --- | --- | --- |
| **Varicella** |  |  |
|  |  |  |
| Varicella without complication | | 052.9^ |
| Varicella with complication | | 052.7^*, 052.8^ |
| Neurological complications | Encephalitis | 052.0^, 049.8, 049.9, 136.9, 294.1, 323.0, 323.4, 323.6, 323.8, 323.9 |
|  | Meningitis | 052.7^*, 047.8, 047.9, 321.2, 321.8, 322.0, 322.9 |
|  | Demyelineating disease | 323.9, 341.0, 341.8, 341.9, 377.3 |
|  | Cerebellar ataxia | 334.3, 334.4 |
|  | Viral infection of the central nervous system | 049.9 |
| Pneumonia/ Pneumonitis | Varicella pneumonitis | 052.1^ |
|  | Pneumococcal pneumonia | 481* |
|  | Streptococcal pneumonia | 482.30-482.39 |
|  | Staphylococcal pneumonia | 482.4 |
|  | Other pneumonia/ pneumonitis | 136.9, 480, 480.8, 480.9, 481*, 482.9, 483.8, 484, 484.8, 485, 486 |
| Septicaemia |  | 038.0-038.3, 038.40-038.49, 038.8-038.9 |
| Other bacterial infections | Toxic shock syndrome | 040.89, |
|  | Pneumococcal infection | 041.06, 041.2, 711.00 |
|  | Streptococcal infection | 034.0, 034.1, 041.00-041.05, 041.09 |
|  | Staphylococcal infection | 041.10, 041.11, 041.19, 711.00 |
| Skin and soft tissue infections | Cellulitis, abscess and erysipelas | 035, 681.0-681.9, 682.0-682.9 |
|  | Impetigo | 684, 686.8 |
|  | Fasciitis | 728.86, 729.4 |
|  | | 370.40, 370.49, 372.00, 372.02, 372.03, 372.05, 372.20, 372.30, 372.33, 372.39 |
| Congenital varicella infection | | 771.8 |
| VZV in pregnancy/ postpartum | | 647.60, 647.63, 647.64 |
| **Zoster** |  |  |
|  |  |  |
| Zoster (without mention of complication) | | 053.9^ |
| Zoster with complications | | 053.7^, 053.79^, 053.8^ |
| Geniculate herpes zoster/ Ramsay Hunt syndrome | | 053.11^ |
| Neurological complications | Encephalitis | 053.19^, 049.8, 049.9, 136.9, 294.1, 323.0, 323.4, 323.6, 323.8, 323.9 |
|  | Meningitis | 053.0^, 047.8, 047.9, 321.2, 321.8, 322.0, 322.9 |
|  | Postherpetic neuralgia | 053.19^ |
|  | Postherpetic polyneuropathy | 053.13^ |
|  | Postherpetic trigeminal neuralgia | 053.12^ |
|  | Viral infection of the nervous system | 053.1^, 053.10^, 053.19^, 049.9 |
| Zoster with auricular involvement | | 053.71^ |
| Zoster with ophthalmic complications | | 053.20-053.29 |
| VZV in pregnancy/ postpartum | | 647.60, 647.63, 647.64 |
| **Immunodeficiency** | |  |
|  |  |  |
|  | Malignancy involving solid organ/ tissue | 140-165, 170-176, 179-199, 573.8 |
|  | Lymphoproliferative malignancy | 200-208 |
|  | Transplantation# | 996.8, V42.0-V42.4, V42.6-V42.9, V58.49 |
|  | Human immunodeficiency virus | 042-044, 294.1,  647.60, 647.61, 647.63, 647.64, 795.8, V02.9, V08 |
|  | Chemotherapy | 277.8, 799.8, V07.3, V07.39, V58.1, V66.1, V66.2, V67.2 |
|  | Radiotherapy | 253.7, 457.1, 558.1, 990, E879.2, V58.0, V67.1 |
|  | Asplenia or other splenic disease | 289.4, 289.5, 759.0, V45.89 |
|  | Other conditions affecting immune system** | 277.8, 279.01-279.09, 279.1-279.9, 282.4, 284, 284.8, 284.9, 288, 288.8, 648.2, 648.23, V02.9 |
| **Pregnancy** |  | 633.8, 634-677, 694.3, 694.4, 760-763, 773.2, 779.9, 799.9, V22-V24, V27-V39, V41.9, V61.7, V65.9 |
|  |  |  |

Remarks:

*^ICD-9-CM codes specific to varicella and zoster*

**An extension code known as Term ID is adopted by the Hospital Authority to supplement the ICD-9-CM codes. Thus the same ICD-9-CM code may represent different description as their Term ID differs*

*#excluding corneal transplantation*

***Including primary immunodeficiency, WBC diseases, aplastic anaemia, thalassaemia major, and other specified diseases with participation of lymphoreticular and reticulohistiocytic tissue*

**Supplementary Table S2. Estimation of proportion seroconverted among vaccinees in different serological surveys**

| **Year of survey** | **Age group** | **Birth cohort^1^** | **Proportion vaccinated^2^** | **Proportion seroconverted due to vaccination^3^** |
| --- | --- | --- | --- | --- |
| 1995 | 1 to 4 | 1991-1994 | 5.7% | 3.7% |
|  | 5 to 9 | 1986-1990 | 0.0% | 0.0% |
|  | 10 to 14 | 1981-1985 | 0.0% | 0.0% |
|  | 15 to 19 | 1976-1980 | 0.0% | 0.0% |
| 2000 | 1 to 4 | 1996-1999 | 9.3% | 6.1% |
|  | 5 to 9 | 1991-1995 | 5.7% | 3.7% |
|  | 10 to 14 | 1986-1990 | 0.0% | 0.0% |
|  | 15 to 19 | 1981-1985 | 0.0% | 0.0% |
| 2005 | 1 to 4 | 2001-2004 | 22.1% | 14.3% |
|  | 5 to 9 | 1996-2000 | 12.7% | 8.2% |
|  | 10 to 14 | 1991-1995 | 5.7% | 3.7% |
|  | 15 to 19 | 1986-1990 | 0.0% | 0.0% |
| 2010 | 1 to 4 | 2006-2009 | 36.1% | 23.4% |
|  | 5 to 9 | 2001-2005 | 24.4% | 15.9% |
|  | 10 to 14 | 1996-2000 | 12.7% | 8.2% |
|  | 15 to 19 | 1991-1995 | 5.7% | 3.7% |

Remarks:

*1. Birth cohort deduced from age group and corresponding survey year.*

*2.Proportion vaccinated was obtained from first dose varicella vaccination uptake in the immunisation coverage surveys on preschool children in 2001, 2003, 2006, 2009, 2012 and 2015, which covered birth cohorts between 1995 to 2011(please refer to Fig 1a of this manuscript). Children of birth cohorts 1991 to 1994 were assumed to have same vaccination uptake as the 1995 cohort (i.e. 5.7%). Individuals born in 1990 or before were assumed to be unvaccinated as varicella vaccine were only available in the private market in 1996.*

*3.* *Sauerbrei et al. compared different laboratory tests for assessing varicella immunity. Blood samples of 57 vaccinees aged 2 to 35 years were obtained 4 to 6 weeks after one or two doses of monovalent varicella vaccine (mVV). 37 (65%) tested positive by a commercially available whole-cell ELISA kit. Numbers in these columns were obtained by multiplying this proportion with the proportion vaccinated.*

**Supplementary Figure S1. Coding practice of A&E attendance and inpatient admission in public hospitals of Hong Kong, 2004/05 to 2013/14. (a) Proportion of A&E attendance coded, (b) average number of codes per A&E attendance, (c) proportion of A&E referred admission coded and (d) average number of codes per A&E referred admission**

| (a) | 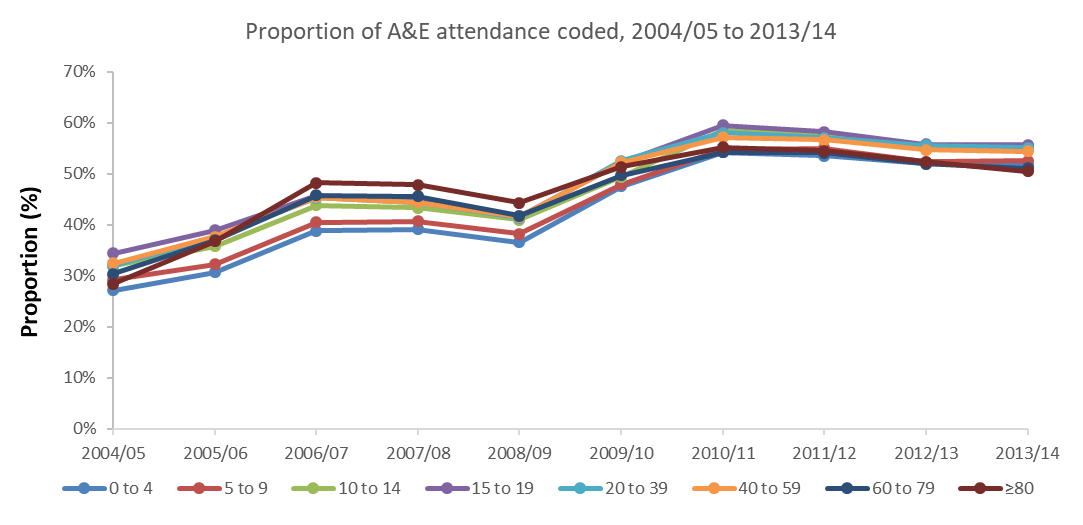 |
| --- | --- |
| (b) | 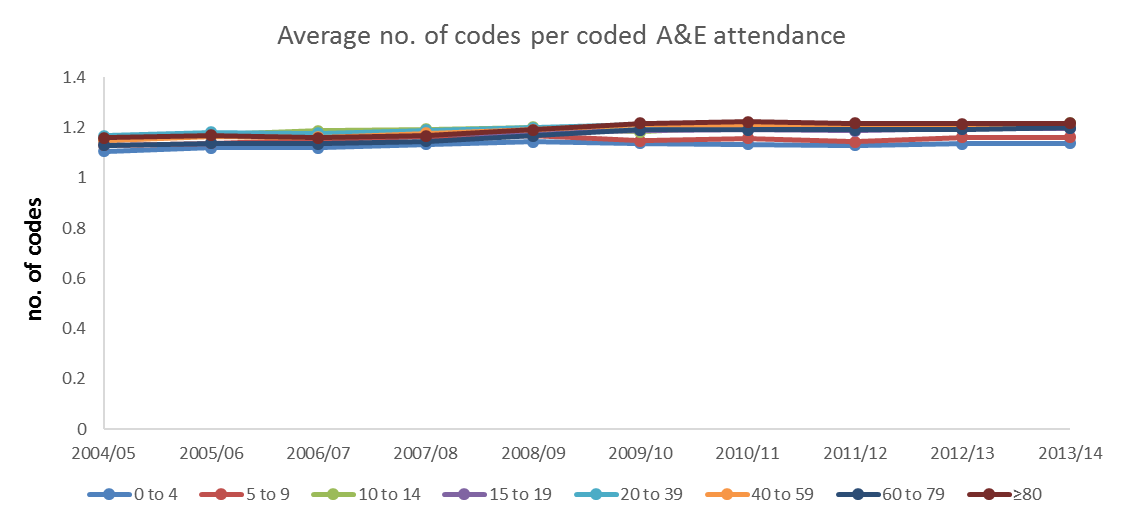 |
| (c) | 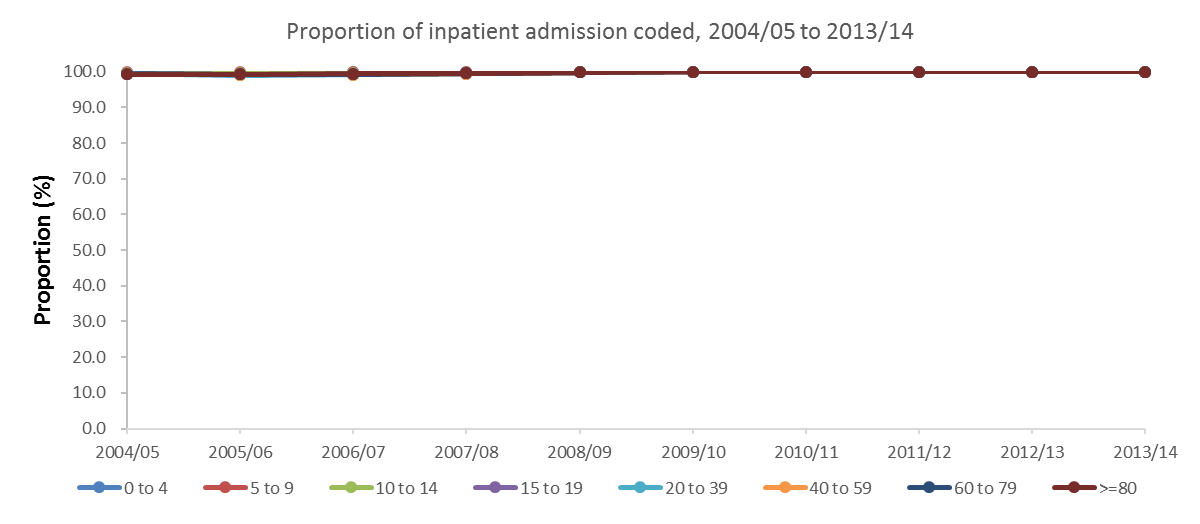 |
| (d) | 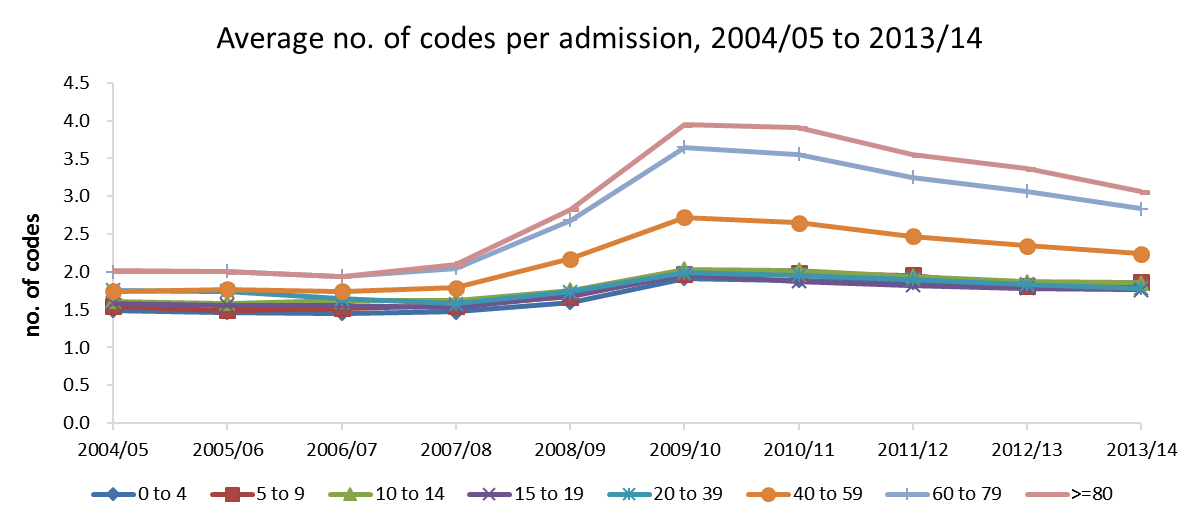 |

**Supplementary Figure S2. No. of enrolment, average no. of enrolment per school and per class in preschools^, primary and secondary schools in Hong Kong, 1990 to 2015 (Source: Education Bureau and Social Welfare Department, Hong Kong SAR Government).**


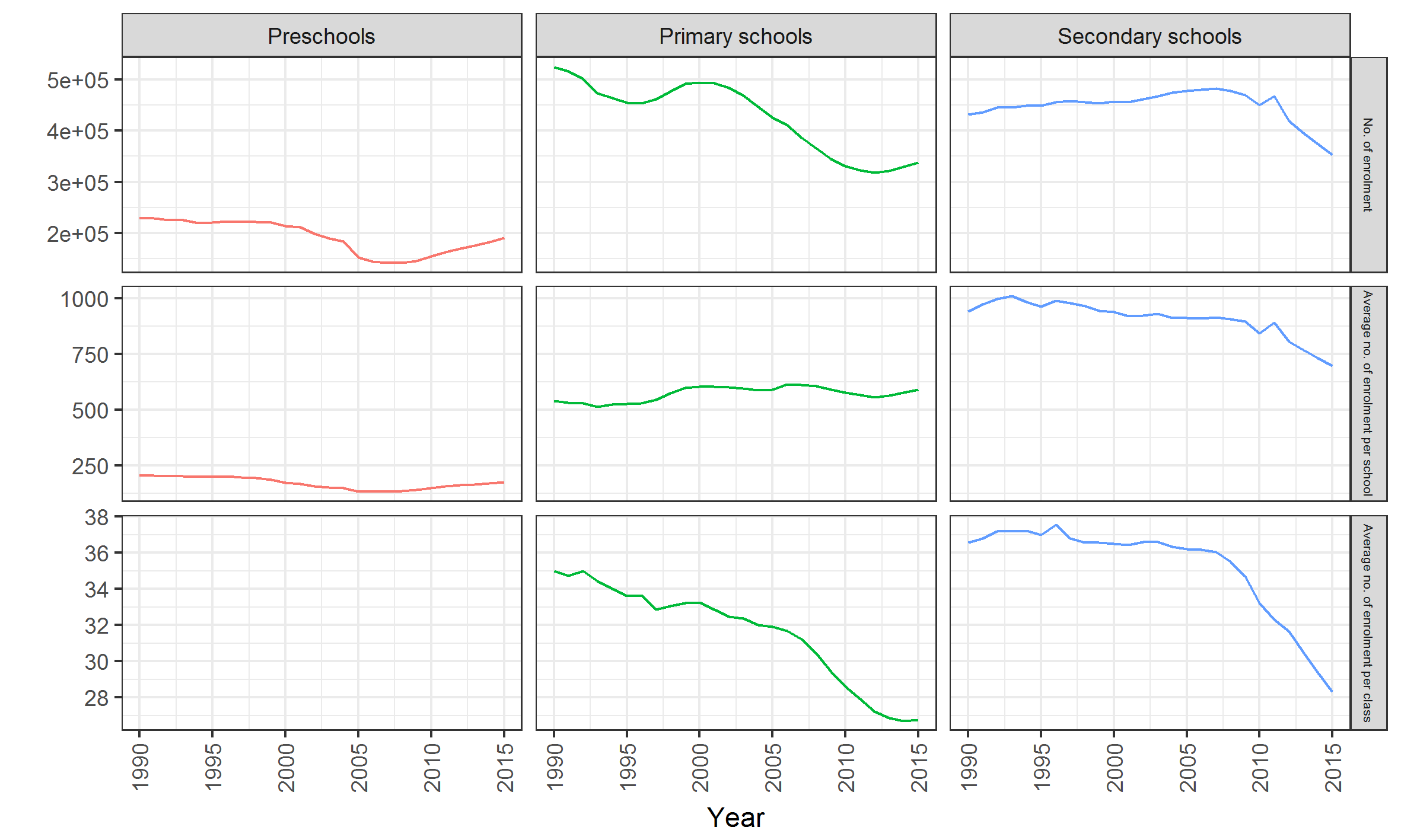


Remarks:

*^Preschools include kindergartens (KG), child-care-centres (CCC) and KG-cum-CCC. Data regarding no. of enrolment per class is not available for preschools.*

**Supplementary Figure S3. Proportion seropositive against varicella antibody and model fitting with sensitivity analysis^**


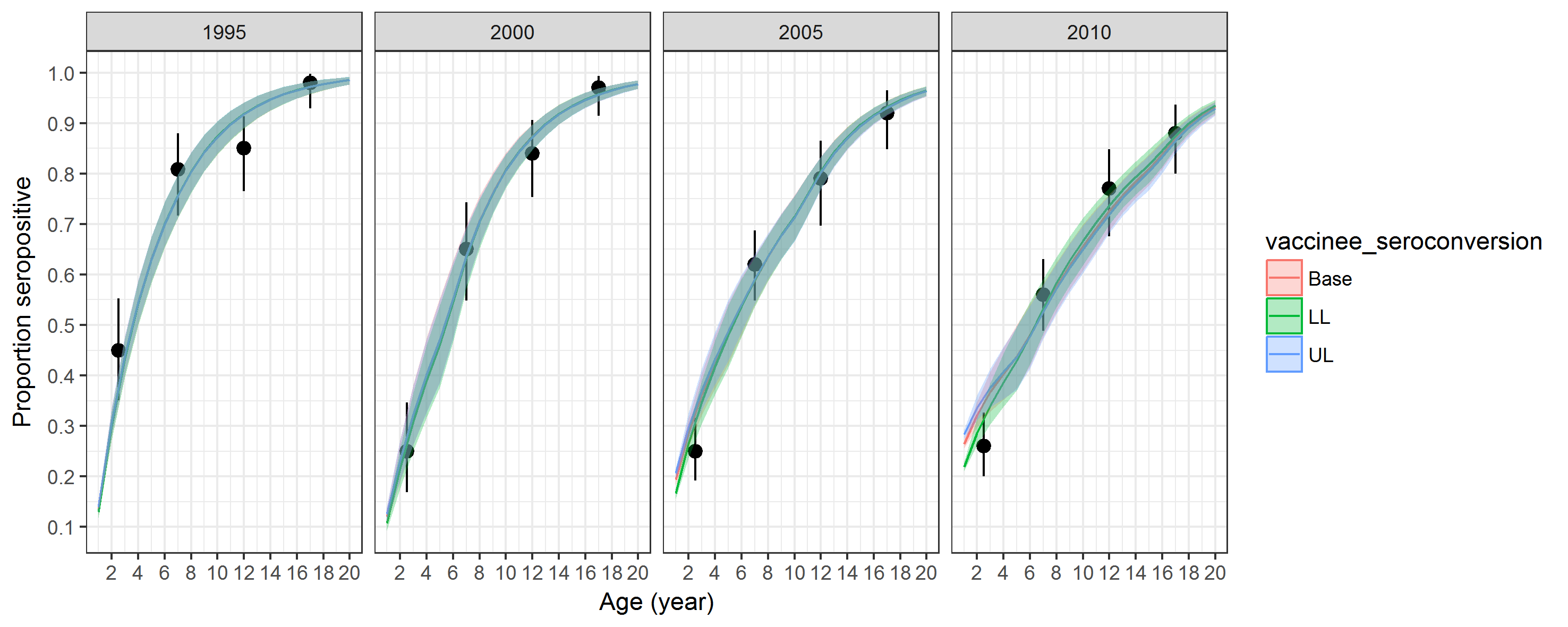


Remarks:

*^Sensitivity analysis was carried out based on the proportion of vaccinee seroconverted and its 95%CI from Sauerbrei et al., for which 37 of 57 (65%) of individuals aged 2 to 35 years were tested positive by a commercially available wc ELISA kit, 4 to 6 weeks after receiving one or two doses of mVV. Base: 65%, lower limit (LL): 51% and upper limit (UL): 77%.*
